# Supplementary material for: Evolutionary analyses of the major variant surface antigen-encoding genes reveal population structure of Plasmodium falciparum within and between continents
Source: PLoS Genet. 2021 Feb 25;17(2):e1009269. doi: 10.1371/journal.pgen.1009269 (PMC7906310; doi:10.1371/journal.pgen.1009269)
Supplement: S1 Text — (PDF) [file pgen.1009269.s001.pdf]

S1 Text. Additional details comparing the JHMM approach to previous methods used for analyzing *var* DBLα population structure (S18 and S19 Figs). A binary presence/absence matrix was built by clustering the global dataset at 96% pairwise sequence identity using the pipeline described in [1]. The resulting matrix indicated which DBLα type (clustered at 96%) was present in each isolate. A principal component analysis (PCA) was then performed (S18 Fig). The first principal component indicated a separation between the South American isolates and the rest of the world. This was consistent with the proportions identified using the JHMM approach, which indicated that the South American isolates were the most distinct. The third and fourth principal components indicated a separation among the African isolates (S19 Fig). By applying t-Distributed Stochastic Neighbor Embedding (t-SNE) [2], we were able to better resolve the global structure from the binary presence/absence matrix (Fig 2B). t-SNE is able to distinguish structure at multiple scales in high dimensional settings whilst preserving local structure. To compare with the approach of Tessema et al. (2015) [3], we built a phylogenetic tree using RAxML [4], based on treating each isolate's binary presence/absence vector as a binary sequence (Fig S8). Finally, to compare with the approach of Rougeron et al. (2017) [5] we used the same binary matrix as input to Admixture in order to estimate underlying historical populations [6] (S9 Fig). Using cross-validation as a measure of clustering error, we found that two latent clusters were most strongly supported (S10 Fig). Whilst approaches based on clustering at a global sequence identity threshold of 96% identified broadly similar structure in the global *var* population, the JHMM approach is better able to disentangle more subtle or distant relationships such as the affinity between species and the relationship between countries within Africa.

To compare with an approach not reliant on clustering at 96% pairwise sequence identity we used the alignment-free Feature Frequency Profile approach of Sims et al. (2009) [7]. Alignment-free algorithms have proved useful in analyzing large complex datasets where recombination, genetic shuffling and other genetic events make generating accurate alignments difficult [8,9]. Using Jensen-Shannon divergence as a distance measure between the resulting k-mer frequency profiles for each isolate, we built a neighbor-joining tree using FastME v2.1.4 [10]. The resulting phylogeny is shown in S11 Fig.

REFERENCES

1. Ruybal-Pesántez S, Tiedje KE, Tonkin-Hill G, Rask TS, Kamya MR, Greenhouse B, et al. Population genomics of virulence genes of Plasmodium falciparum in clinical isolates from Uganda. Sci Rep. 2017;7: 11810. doi:10.1038/s41598-017-11814-9
2. van der Maaten L, Hinton G. Visualizing Data using tSNE. J Mach Learn Res. 2008;9: 2579–2605.
3. Tessema SK, Monk SL, Schultz MB, Tavul L, Reeder JC, Siba PM, et al. Phylogeography of var gene repertoires reveals fine-scale geospatial clustering of Plasmodium falciparum populations in a highly endemic area. Mol Ecol. 2015;24: 484–497. doi:10.1111/mec.13033
4. Stamatakis A. {RAxML} version 8: a tool for phylogenetic analysis and post-analysis of large phylogenies. Bioinformatics. 2014;30: 1312–1313.
5. Rougeron V, Tiedje KE, Chen DS, Rask TS, Gamboa D, Maestre A, et al. Evolutionary structure of Plasmodium falciparum major variant surface antigen genes in South America : Implications for epidemic transmission and surveillance. Ecol Evol. 2017;7: 9376–9390. doi:10.1002/ece3.3425
6. Alexander DH, Novembre J, Lange K. Fast model-based estimation of ancestry in unrelated individuals. Genome Res. 2009;19: 1655–1664.
7. Sims GE, Jun S-R, Wu GA, Kim S-H. Alignment-free genome comparison with feature frequency profiles (FFP) and optimal resolutions. PNAS. 2009;106: 2677–2682.
8. Song K, Ren J, Reinert G, Deng M, Waterman MS, Sun F. New Developments of Alignment-Free Sequence Comparison: Measures, Statistics and next-Generation Sequencing. Brief Bioinform. 2014;15: 343–353.
9. Bonham-Carter O, Steele J, Bastola D. Alignment-Free Genetic Sequence Comparisons: A Review of Recent Approaches by Word Analysis. Brief Bioinform. 2014;15: 890–905.
10. Lefort V, Desper R, Gascuel O. FastME 2.0: A Comprehensive, Accurate, and Fast Distance-Based Phylogeny Inference Program. Mol Biol Evol. 2015;32: 2798–2800.
