# Supplementary figures and images for: Evolutionary analyses of the major variant surface antigen-encoding genes reveal population structure of Plasmodium falciparum within and between continents
Source: PLoS Genet. 2021 Feb 25;17(2):e1009269. doi: 10.1371/journal.pgen.1009269 (PMC7906310; doi:10.1371/journal.pgen.1009269)

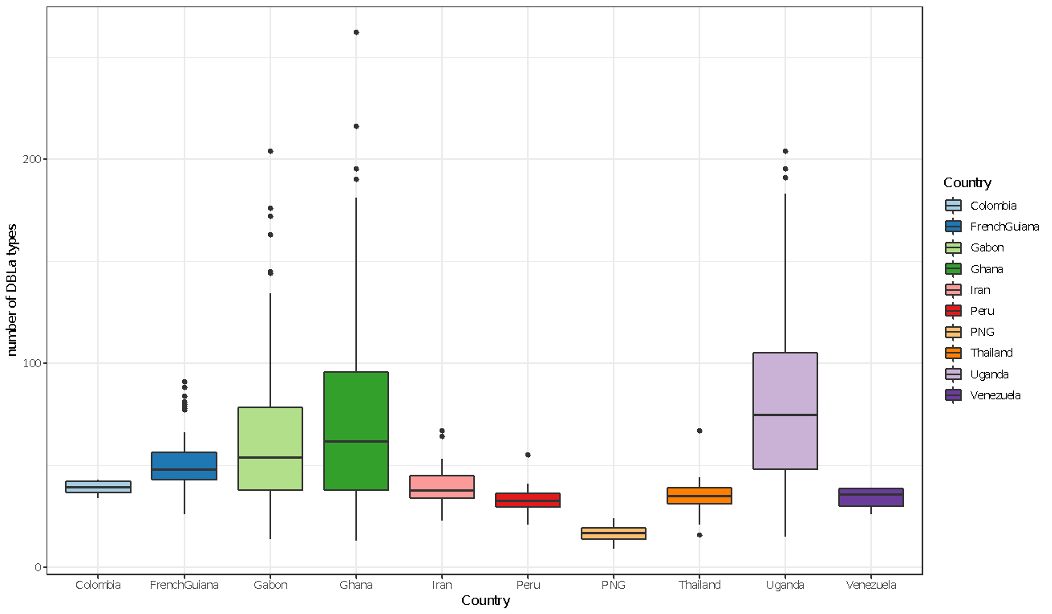

Supplement: S1 Fig — The African countries have significantly higher numbers of types indicating the higher prevalence of multiple-genome infections in Africa. Isolates with less than 20 DBLα types have been excluded. (TIF) [file pgen.1009269.s002.tif]

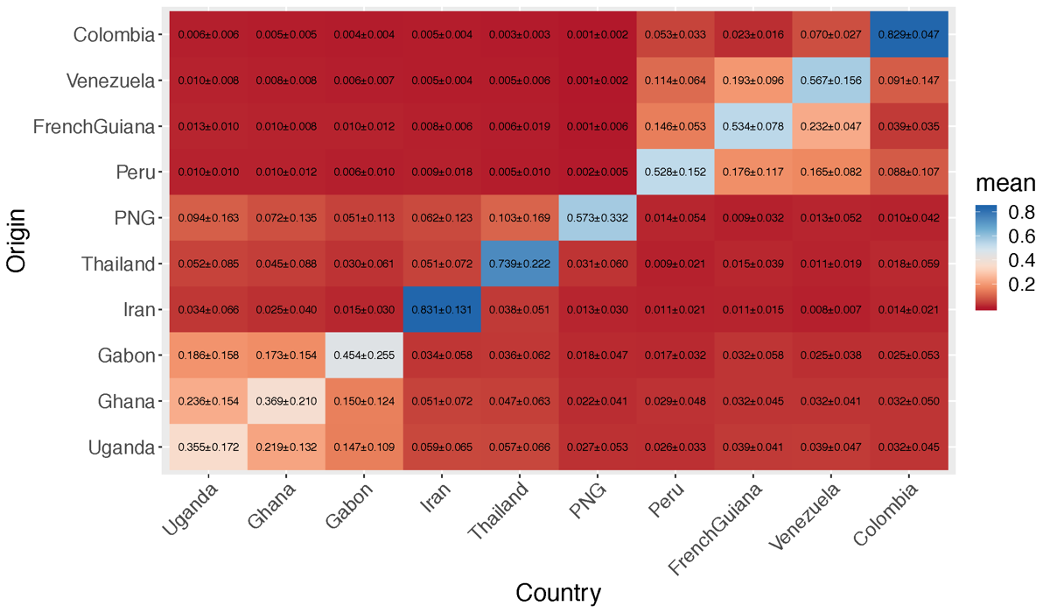

Supplement: S2 Fig — The relationship between countries mirrors that seen in Fig 4B indicating that the result is robust to the sampling coverage for each country. (TIF) [file pgen.1009269.s003.tif]

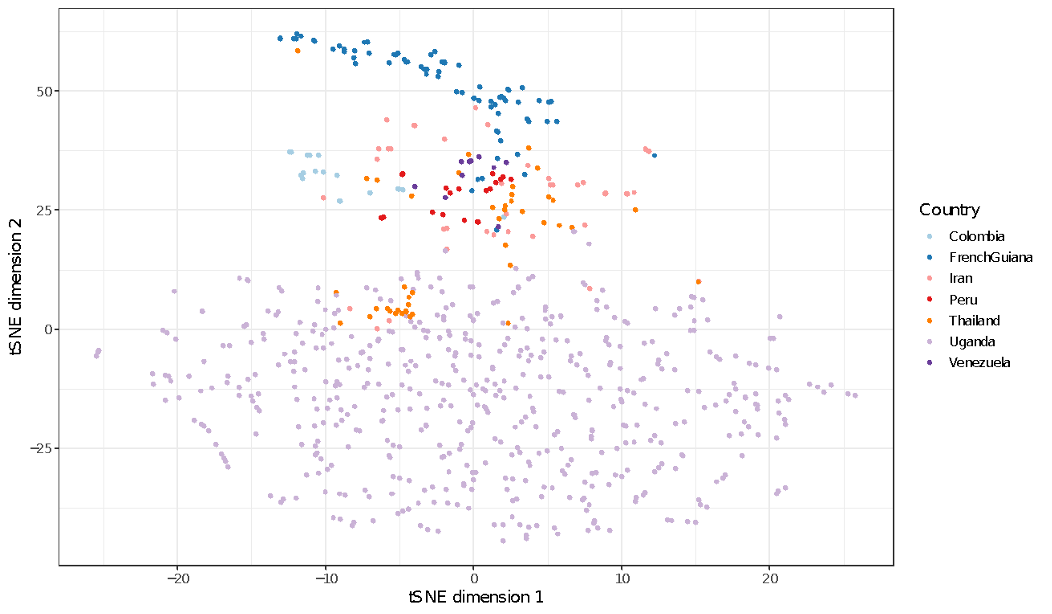

Supplement: S3 Fig — (TIF) [file pgen.1009269.s004.tif]

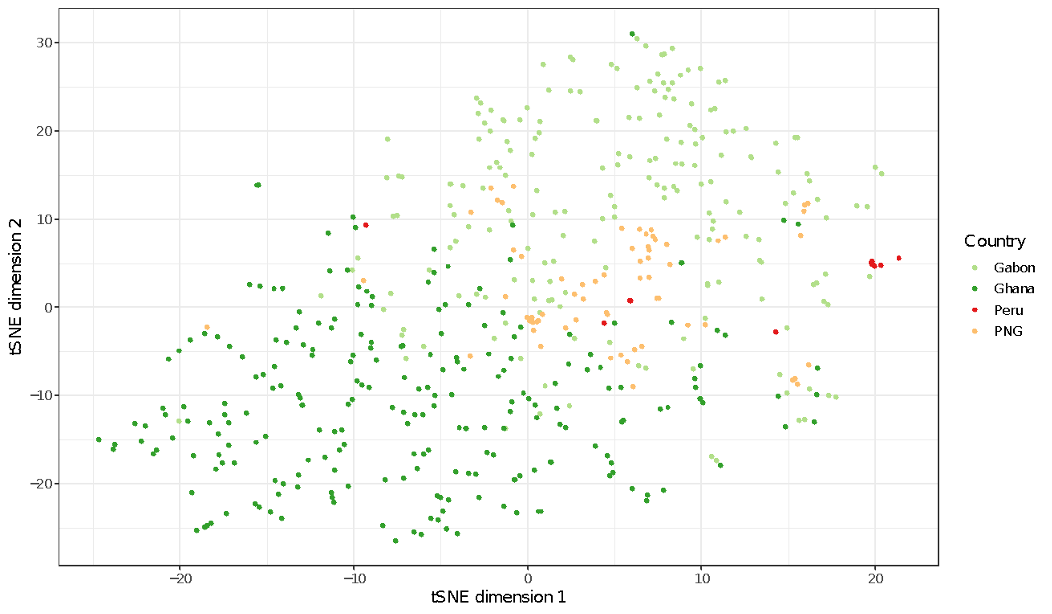

Supplement: S4 Fig — (TIF) [file pgen.1009269.s005.tif]

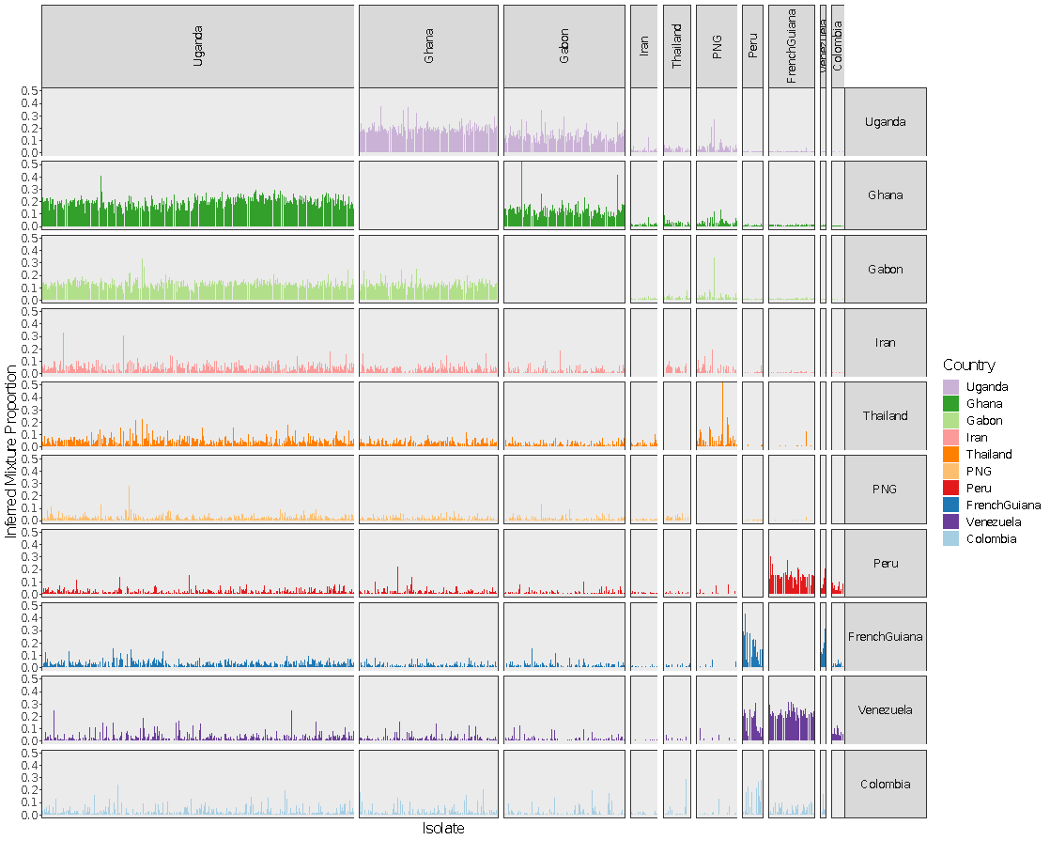

Supplement: S5 Fig — An isolate’s proportions are represented as a column in the graph where a column would add to one if self-matching was included. The African isolates preferentially match with other African populations. Similarly, South American isolates match nearly entirely with other South American populations. PNG, Thailand and Iran are more closely related to the African isolates with the PNG isolates reporting a larger proportion of matching to Iran and Thailand than isolates from other countries. A small number of isolates with matching profiles that are distinct from other isolates within the same population may represent more recent migrations. (TIF) [file pgen.1009269.s006.tif]

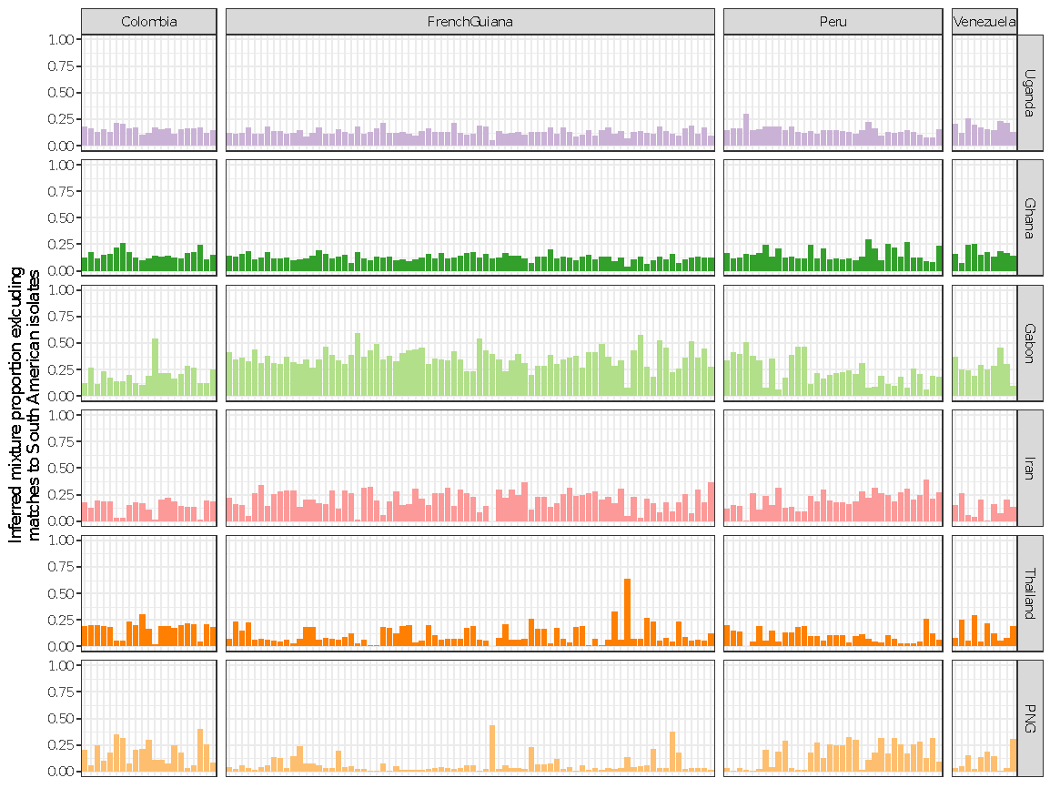

Supplement: S6 Fig — This prevents the algorithm from assigning ancestry to other South American isolates and thus allows us to focus on the relationships with the remaining countries. The proportions indicate no strong link between any of the South American countries and any one African country. (TIF) [file pgen.1009269.s007.tif]

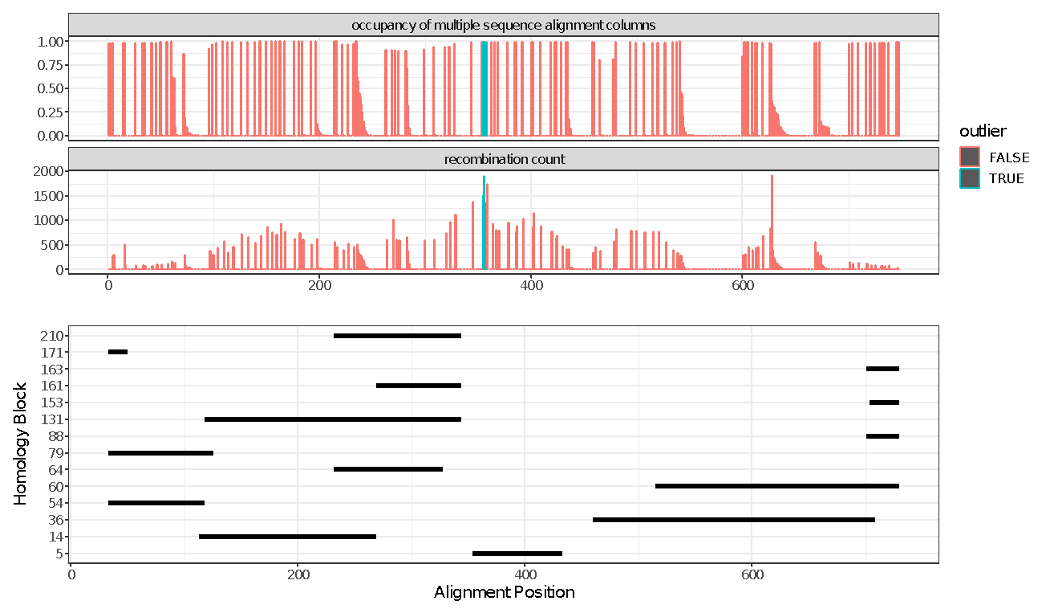

Supplement: S7 Fig — The symmetry between the two plots indicates that recombination occurs throughout the DBLα tag with only one multiple sequence alignment column found to be an outlier. An alignment of the relevant homology blocks from Rask et al. (2010) [6], is given below the two bar plots. (TIF) [file pgen.1009269.s008.tif]

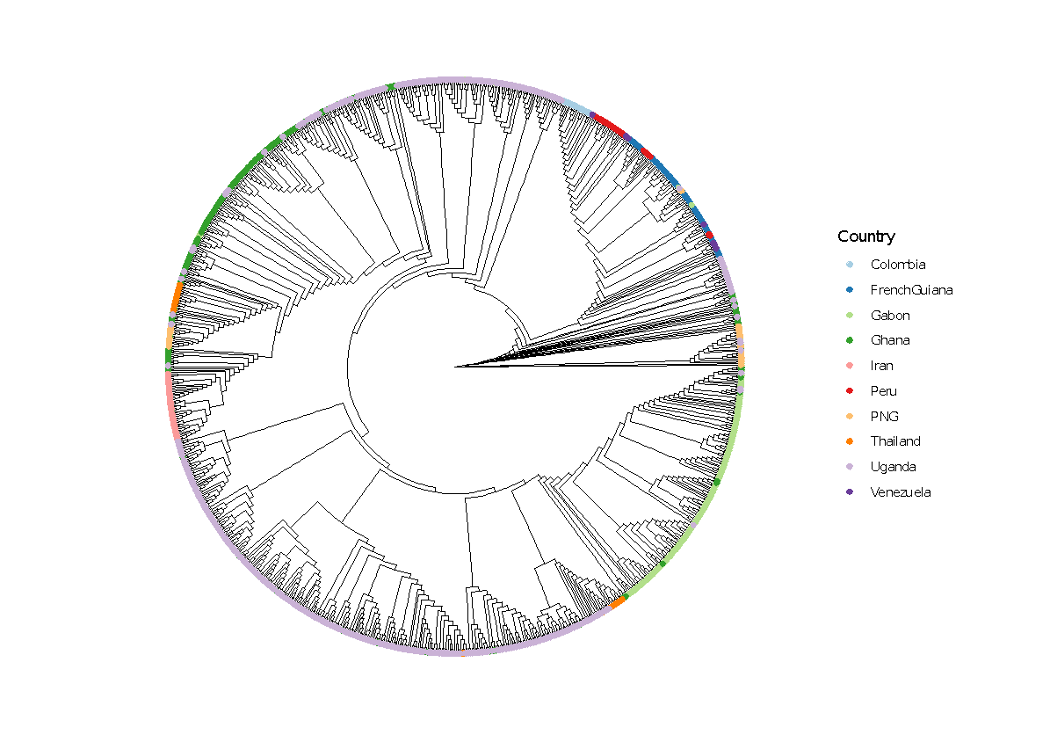

Supplement: S8 Fig — The population structure evident in the t-SNE plot is reproduced in this analysis. Peru is split into two populations and Ghana is more distinct from Uganda than the FFP and Admixture analysis. Colombia is found to be closer to the other South American isolates in this analysis. (TIF) [file pgen.1009269.s009.tif]

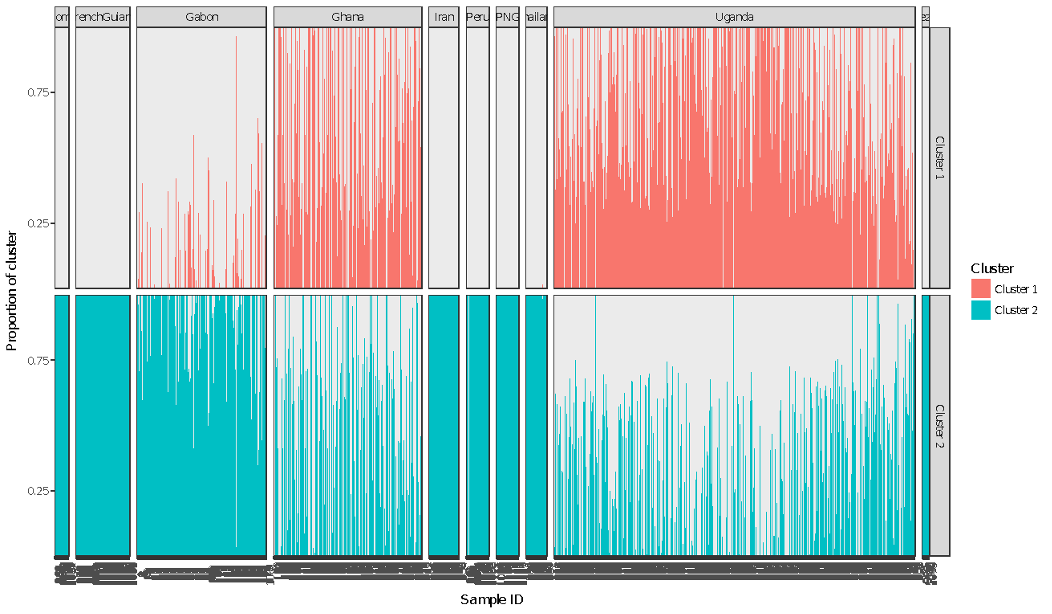

Supplement: S9 Fig — An isolate is represented as a single haploid chromosome with the alternative allele indicating that a DBLα type is present in that isolate. The separation between African and the non-African populations is clear. (TIF) [file pgen.1009269.s010.tif]

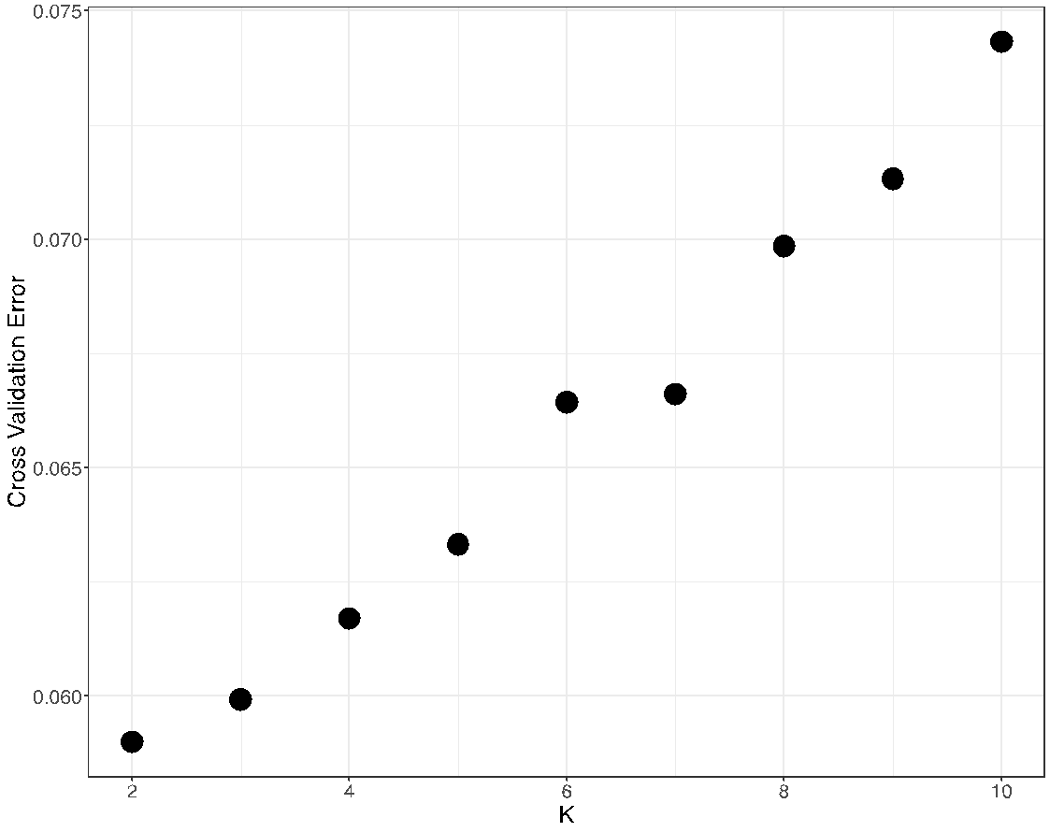

Supplement: S10 Fig — (TIF) [file pgen.1009269.s011.tif]

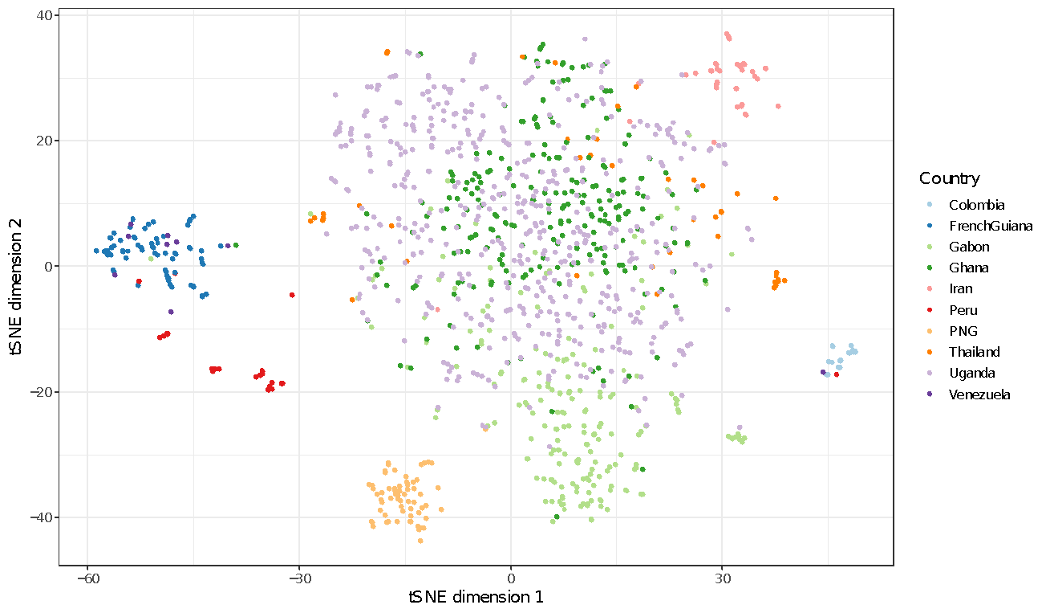

Supplement: S11 Fig — Whilst clustering by country is evident, the resolution is poorer than was achieved using the binary presence/absence-based distance. (TIF) [file pgen.1009269.s012.tif]

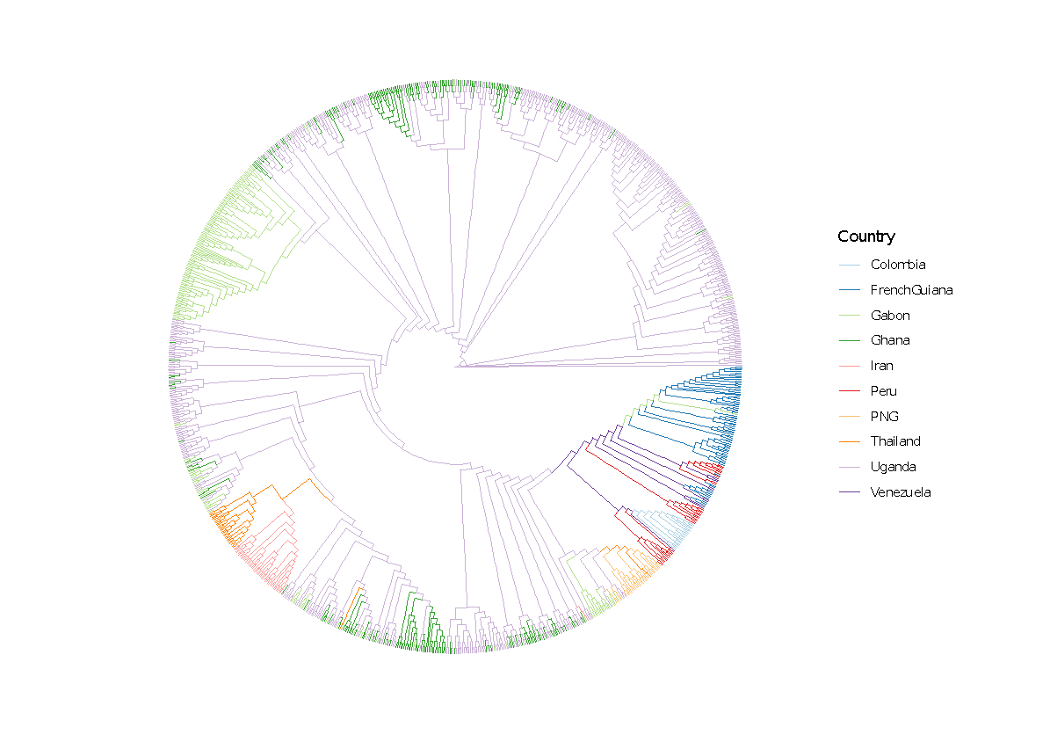

Supplement: S12 Fig — The tree was constructed using the default FastMe v2.1.4 [70] method from a distance matrix generated using the Feature Frequency Profile (FFP) approach of Sims et al. (2009) [52] with a k-mer length of 20. The country level population structure is evident; however, Ghana is less separated from Uganda than in the t-SNE and JHMM approaches. (TIF) [file pgen.1009269.s013.tif]

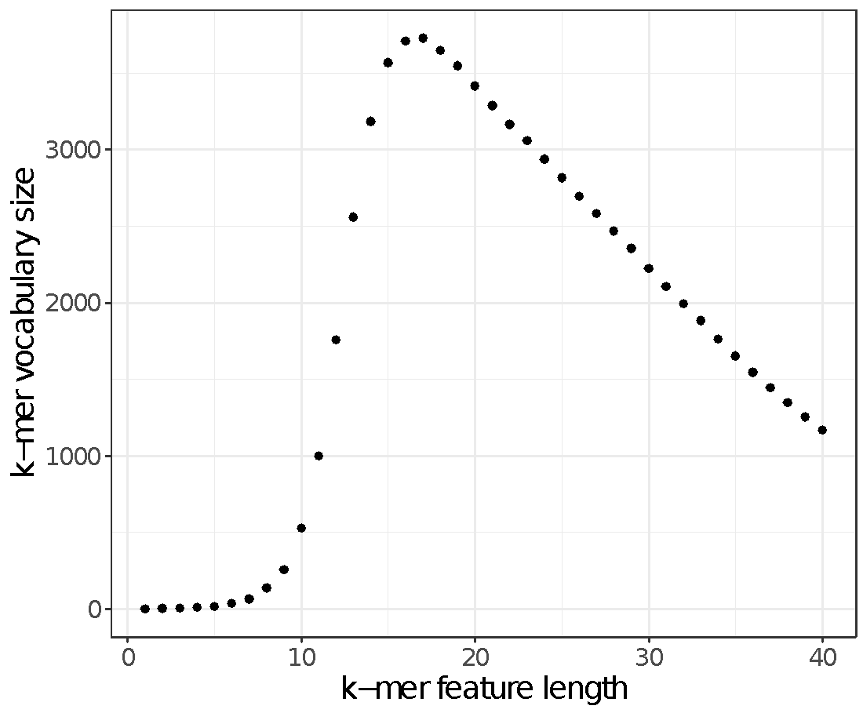

Supplement: S13 Fig — This can be used to set a lower bound for the choice of k-mer length by looking for the maximum of the vocabulary size [52]. (TIF) [file pgen.1009269.s014.tif]

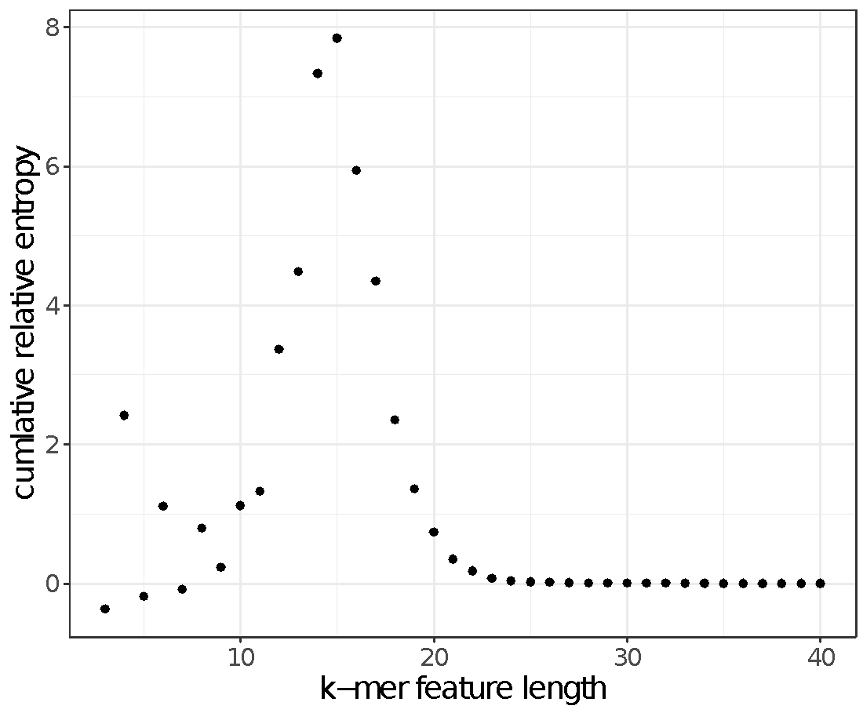

Supplement: S14 Fig — See Sims et al. (2009) [52] for a detailed description. (TIF) [file pgen.1009269.s015.tif]

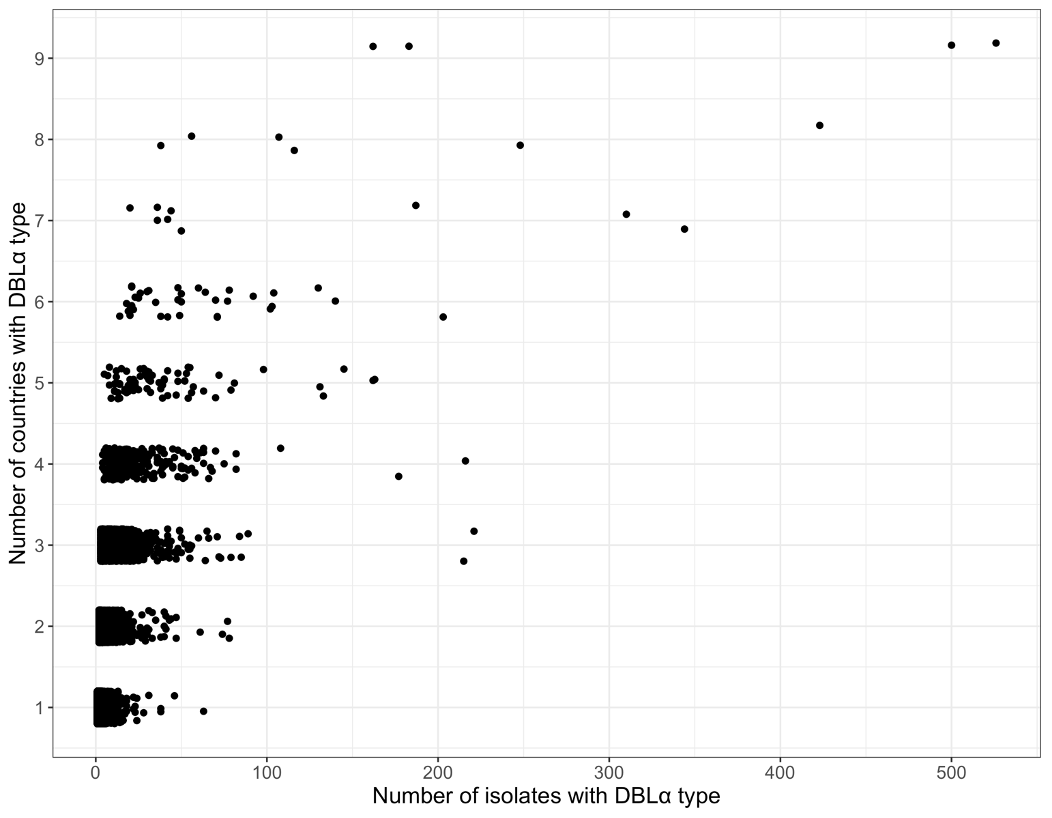

Supplement: S15 Fig — The high density of the types seen in only one country is driven by the large number of unique DBLα types identified. (TIF) [file pgen.1009269.s016.tif]

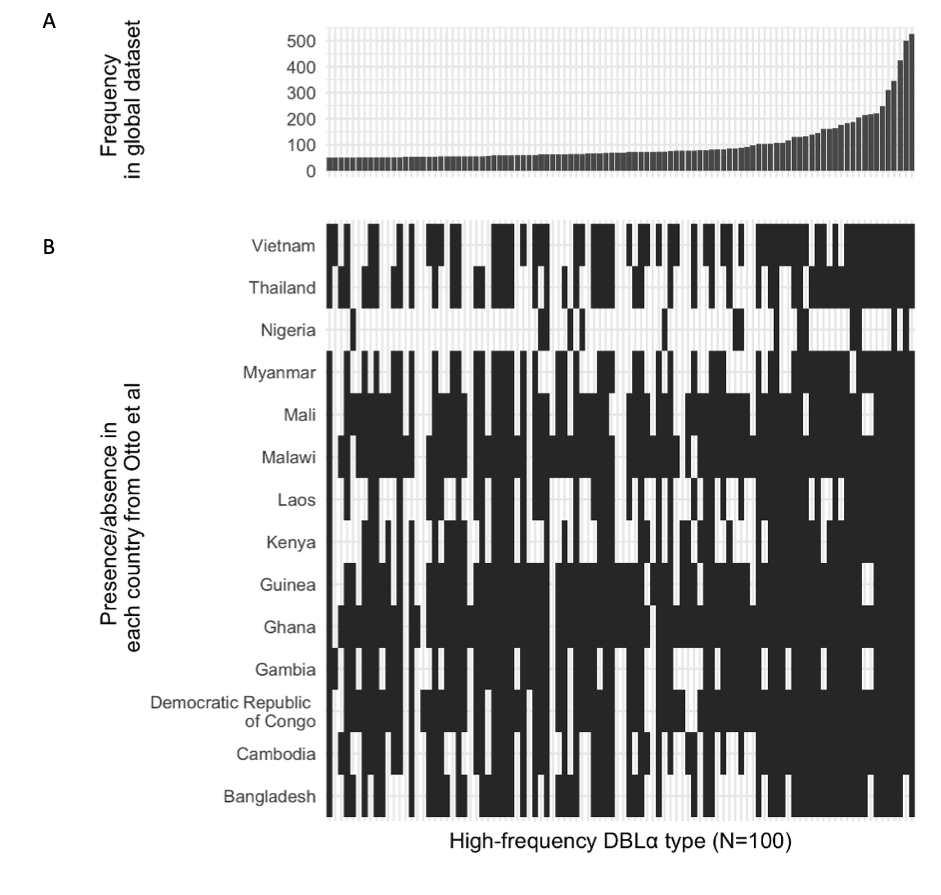

Supplement: S16 Fig — A. The frequency of the 100 high-frequency DBLα types in our global dataset (i.e., the number of P. falciparum isolates each type was observed in out of 1,248 isolates). B. The presence/absence of each high-frequency type after searching for them in the independent assembly of var genes from Otto et al. (2019) [5], where black denotes presence and white denotes absence stratified by country of origin. The order of DBLα types along the x-axis is the same in both A and B. (TIF) [file pgen.1009269.s017.tif]

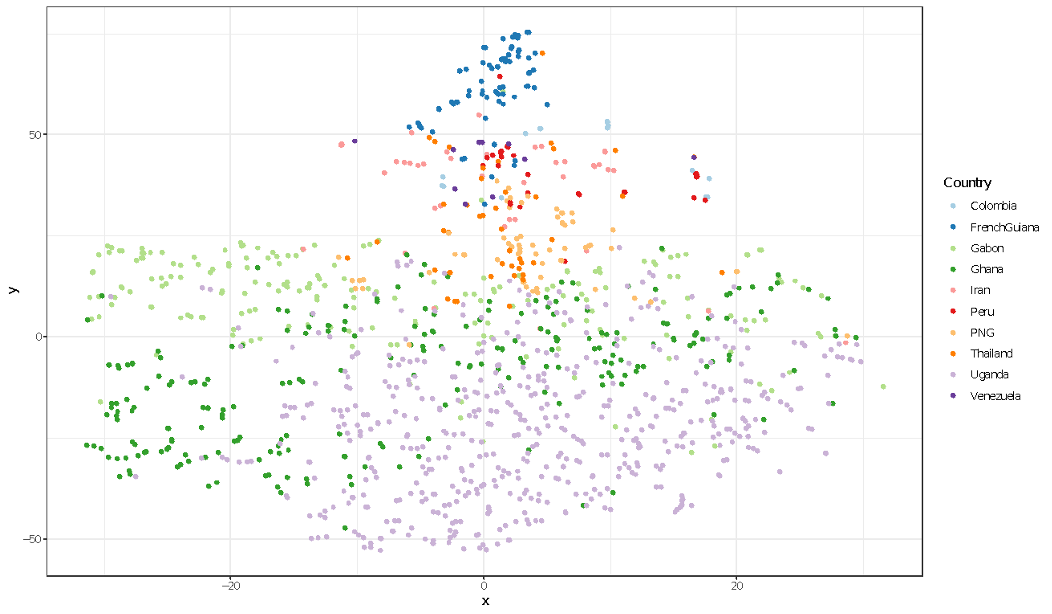

Supplement: S17 Fig — Whilst the clustering is less defined than Fig 2B, the overall grouping by country is still clearly evident suggesting that the result is robust to the commonly used practice of filtering out isolates with less than 20 DBLα types. (TIF) [file pgen.1009269.s018.tif]

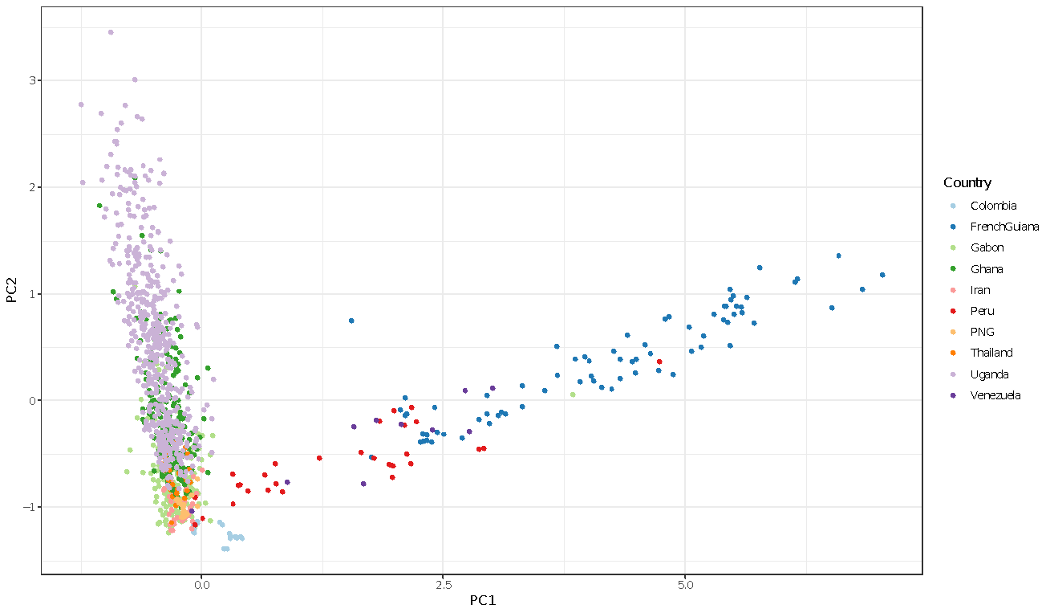

Supplement: S18 Fig — A clear separation between the South American isolates is apparent. (TIF) [file pgen.1009269.s019.tif]

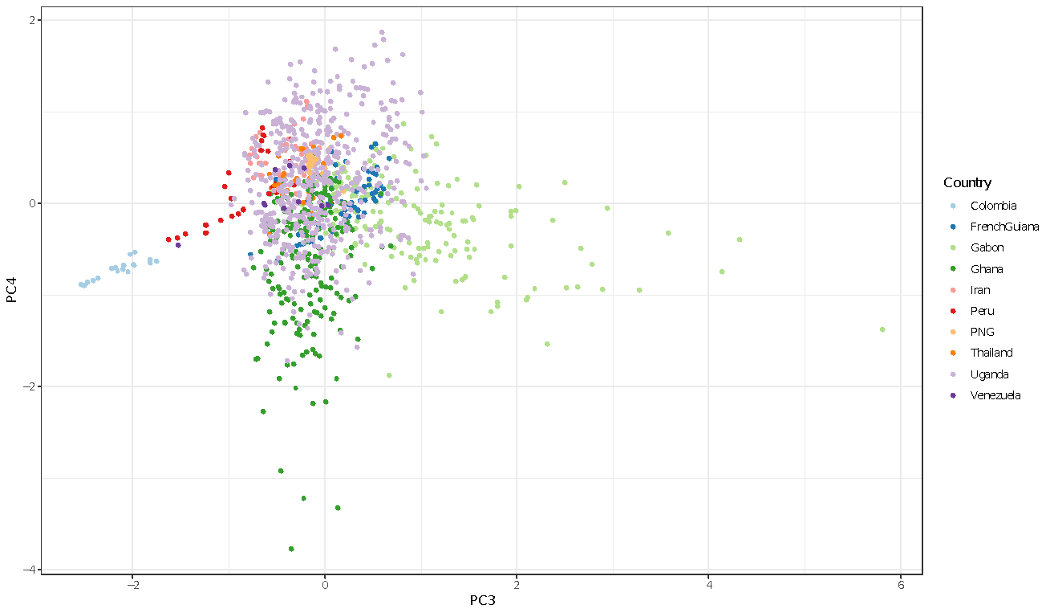

Supplement: S19 Fig — Although there is still significant overlap, the separation between the African countries is shown. A much clearer distinction was found in the t-SNE analysis. (TIF) [file pgen.1009269.s020.tif]
