# Supplementary material for: Evolutionary analyses of the major variant surface antigen-encoding genes reveal population structure of Plasmodium falciparum within and between continents
Source: PLoS Genet. 2021 Feb 25;17(2):e1009269. doi: 10.1371/journal.pgen.1009269 (PMC7906310; doi:10.1371/journal.pgen.1009269)
Supplement: Attachment — Submitted filename: Response-PGENETICS-D-20-00892_Final.docx [file pgen.1009269.s025.docx]

**Tonkin-Hill et al. (PGENETICS-D-20-00892)**

**RESPONSE TO REVIEWERS**

## **Reviewer #1**

**In this paper, the authors analyse the global diversity of the DBLa domains, from the polymorphic var gene family in P. falciparum. It is a huge body of work. Published data are complemented with novel sequences, and an HMMer approach was used to find distant similarity between the domains. These findings allowed the author to find population-specific signatures, which was thought to be not possible due to the sheer diversity of the var genes. But there must be immune pressure to form "populations". The authors use this to confirm existing population structure, analysis samples from related chimpanzee samples and speculate about the out of Africa hypothesis. Overall I enjoyed reading the manuscript, and I have following points to discuss**

1. **The JHMM method is key to this paper. And it is described in the paper, but I would not be able to implement it from the article. Also, I am not sure if it is on the git-hub page, which is not very self-explanatory - but helpful. So I wonder if the author could put the command how they run the program in the methods to be able to reproduce the data and make it accessible to others in the field.**

The command to run the JHMM method is indeed included in the GitHub page (<https://github.com/gtonkinhill/global_var_manuscript/blob/master/supplementary_methods_2.Rmd>) but we agree with the reviewer that we could make this clearer in the manuscript. We have added the following information to the methods in LINE 547 to make this clearer: *“The exact commands used to run the Mosaic algorithm are provided in the “supplementary_methods_2.Rmd” file available in the GitHub repository along with the Mosaic source code.”*

2. **One argument of this study is to use PCR of DBLa to do population studies. How does that compare to SNP arrays or other PCR based methods, in terms of time and cost?**

Different approaches have been used for population genetic studies, including SNPs, microsatellite markers (MS), and whole genome sequencing (WGS). Although these methods have been shown to be informative, depending on the research question, each has limitations. Our comment about cost effectiveness on LINE 113 relates to comparisons with WGS and the bioinformatic assembly of whole *var* genes compared to our *var* typing approach to provide geographic information. WGS is currently still too expensive (~$85 USD per isolate) for large field-based studies and given that high sequencing coverage is required, working with low-density, multi-genomic *P. falciparum* isolates continues to be impractical. From our previous publications, as well the current manuscript we have shown that *var* DBLα typing is a sensitive high throughput approach for analysing low density multi-genome (i.e. MOI ≥ 2) infections where the estimated cost, including reagents and sequencing, is ~$20 USD per isolate.

Although the *var* typing approach is similar in terms of time and costs to some of the newer MS/SNP assays, including molecular inversion probes (~$15 USD per isolate) and microhaplotypes (~$45 USD per isolate), our *var* typing approach has a number of key advantages as a surveillance tool across a variety of epidemiological settings. Firstly, the *var* typing workflow is a straight forward high throughput approach that has been shown to work on isolates collected globally from both clinical and low-density asymptomatic *P. falciparum* infections. Secondly, the *var* genotyping approach involves a single PCR per isolate using a pair of universal DBLα primers (Taylor et al. (2000)) that can reliably fingerprint the ~50-60 DBLα loci per *P. falciparum* genome. As *P. falciparum* infections in high-transmission settings are often composed of multi-genomes (i.e. MOI ≥ 2), the more than 60 unique DBLα loci in these infections can be typed using this *var* PCR and sequencing approach (range: ~50 - 800 DBLα loci per isolate). For MS and SNPs, despite improvements in the approaches available for haplotype reconstruction, it is still difficult to compare isolates composed of multi-genome infections using these methods. Thus, using *var* typing we have shown that you can readily analyse and compare all isolates, regardless of their MOI, using our published analytical methods and bioinformatic pipelines.

Finally, and perhaps most importantly, we propose the use of *var* typing in relation to not only geographic population structure but potentially also immune selection as the *var* genes encode the major surface antigen. In the introduction we liken this to using the spike protein of SARS-CoV-2 or influenza haemagluttinin to follow the transmission dynamics of these pathogens in relation to variant-specific immunity by defining the geographic location of sequences. In contrast, SNPs and MS are considered neutral markers and do not specifically track frequency dependent selection driven by variant specific immunity. Hence the benefit of comparing the effectiveness of typing neutral markers (i.e. SNPs, MS) vs markers under immune selection has to be considered in relation to the questions being asked. We would propose that these various methods of surveillance are complimentary.

3. **I have issues with the use of herd immunity as a term for var genes. Although the parasite is around for so long, no herd immunity was seen and so far all ideas to generate vaccines based on var genes failed. Could you please comment?**

The term is accurate if you believe that immunity to *P. falciparum* blood stages is predominantly variant-specific. Induced infection experiments strongly support this view as does the fact that adults do not acquire immunity to infection due to extensive diversity of PfEMP1 variants. Consequently, we are using the term herd immunity in a manner akin to how variant-specific immunity to influenza is viewed by identifying sequence variation in haemagluttinin genes to identify new “strains”. Put simply “if a variant is not there you can’t be immune to it”. Experimentally this means that you sequence first and this informs measuring herd immunity by providing the amino acid sequence to measure variant specific antibodies. We have further explained our intent that you have to have the sequences first in order to measure variant-specific immunity in the case of PfEMP1. Alyssa Barry has pioneered this approach to DBLα sequences (Barry et al 2011, https://doi.org/10.1074/mcp.M111.008326).

The reviewer is correct that the major variant surface antigen is generally not considered a vaccine candidate due to the extreme diversity. The exception is targeting sequences that are associated with severe disease for vaccine development. Instead, variant-specific immunity to this antigen is considered a major driver of the transmission dynamics of *P. falciparum* as it regulates the persistence of blood stages in the circulation of the human host. Hence surveillance is about transmission dynamics not vaccine candidate surveillance. Whilst we have not measured herd immunity in this study, there are multiple examples of immunity to PfEMP1 variants in natural populations. The original work by Marsh and Howard (doi: 10.1126/science.2417315) and the senior author Karen Day as Karen Forsyth (doi: 10.4269/ajtmh.1989.40.344) and Gupta et al (1994) showing exposure dependent acquisition of variant-specific immunity. This early work was then followed up by a large body of work done by Peter Bull and colleagues from Kilifi (e.g. Bull 1998 Nature Med, doi: 10.1038/nm0398-358). Of particular relevance to our use of the term is the work of Alyssa Barry looking at immunity to DBLα domains cited in the manuscript (Barry 2011, doi: 10.1074/mcp.M111.008326).

We concede that not all malariologists have the same view of the importance of variant specific immunity so have removed herd immunity and replaced it with “population immunity” or “variant-specific immunity” in context throughout the manuscript (e.g. LINE 90: “*Analysis of the population structure of the var genes encoding PfEMP1 is thus important for the control and prevention of the disease as well as for the design of any vaccine targeting PfEMP1 based on an understanding of* ***variant-specific*** *immunity*”)

4. **It has recently shown that P. gaboni does not have functional DBLa domains, but these are pseudonised domains, which also explains the low number. Should they not be excluded from the analysis?**

We have updated the analysis of non-human *Plasmodium* species to focus on the DBLα domain sequences of *P. billcollinsi, P. praefalciparum and P. reichenowi* from Otto et al. (2019). These assemblies were not available when this work was first carried out but as the reviewer states there are now high quality PacBio assemblies of these species. We thus felt it was appropriate to redo this analysis using the more accurate genome assemblies. The updated Figure 1 includes this analysis and draws similar conclusions.

5. **I like the tSNE plot but struggle with the binary presence/absence. In Fig 2B, some populations have a very different pattern. Could it be that there are sampling biases like two samples came from related infections that bias this analysis? Would it not be better to look for a blast based similarity matrix and do the TSNE plot from that matrix? So I cannot understand that Colombia has a similar pattern than Ghana, and they are also more close.**

Whilst we cannot completely exclude the possibility of a small number of recent transmission chains leading to some points being grouped together, these samples were collected from multiple distant sites within countries over a period of years. Thus, it is highly unlikely that the structure we are observing could be explained by recent transmission.

The curved nature of the South American clusters in the t-SNE plot is likely the result of the higher level of conservation in the *var* repertoires of these isolates. In general, it is important to not to overinterpret the shapes within tSNE plots as the algorithm is stochastic and such shapes are sensitive to the parameters used. Fig S17 indicates that slight changes, whilst maintaining the overall country level structure, can lead to different cluster shapes. We repeated this analysis using a BLAST based distance defined in Kelly et al. (2013) (<https://doi.org/10.1371/journal.pone.0058537>). This provided a similar, but less well resolved clustering and is more similar to FFP based approach. Whilst we agree that binary presence/absence based methods may miss some signal from rarer DBLα sequences, we feel that the JHMM provides the best solution to this problem.

To better describe these points, we have added the following text at LINE 248: *“This clustering is unlikely to be the result of recent transmission as these isolates were taken from multiple geographically distant sites within countries over a period of years (Table 1). The different cluster shapes for each country is likely a result of the differences in the level of conservation of the respective DBLα. Such shapes are also sensitive to the parameter choices of the t-SNE algorithm and should not be overinterpreted.”.*

As well as a t-SNE plot derived from the BLAST based distance matrix as a supplementary Figure (Fig S11) at LINE 365: *“We also considered using a BLAST based distance matrix in place of the binary presence/absence matrix, prior to visualization with t-SNE but found it provided a poorer resolution of the global population structure (Fig S11).”*

6. **Due to the Jumping HMM, the base information gets lost. Would it be possible to generate like GSEA like plots, where 10 DBLa domains get taken, and then the hits get ordered by similarity and coloured below by country as a bar code? This would give the reader a better understanding of how clear the clustering per country/region is based on individual DBLa. Ten randomly picked domains from Pf3D7 could do this plot?**

As GSEA plots are usually used in the analysis of gene expression data we are not certain of how they could be applied to this situation. This suggestion seems to assume that the algorithm provides a set of “best hits”. Whilst the viterbi algorithm could be used as part of the JHMM to identify the best hits we instead make use of the forward-backward algorithm. This gives a posterior likelihood of a match between each base in the query sequence and every base in the database. As a result, it does not produce a set of hits that could easily be translated into a plot of individual DBLα types.

7. **Line 246, I cannot follow how the regression addresses the multiplicity of infections.**

To help make this section easier to follow we have added the following text to LINE 273 to provide some extra intuition: “*While we do not infer the multiplicity of infection for each isolate directly, the number of DBLα types was found to be a weaker predictor of an isolate’s country of origin than the inferred mixture proportions. After accounting for the inferred mixture proportions, the number of DBLα types was not found to be significantly associated with an isolate’s country of origin (all p > 0.97). This is consistent with the large overlap in the distributions of the number of DBLα types by country shown in Fig S1. If the observed signal was driven entirely by the overall number of types we would expect to see similar levels of overlap in both the t-SNE plot (Fig 2B) and the JHMM mixtures (Fig 2C), which is not the case.”*

8. **I was wondering if the JHMM could bring more population resolution in West Africa. I agree with the authors that so far their approach confirmed existing population structure. But SNP based methods struggle to find differences in West Africa. Would it be possible to use the DBLa from reference 34 and look for novel structure?**

Whilst we are able to see clear structure between the Ghanaian and Gabonian isolates we were unable to identify clear structure within these countries. We agree that it would be interesting to further investigate country level structure within the isolates of Otto at al. (2019) however we believe that this is outside the scope of our current manuscript. We agree that a future project could use DBLα sequences in comparison to the whole *var* sequences from Otto et al. (2019) to investigate population structure at a finer resolution. If DBLα tells the structure story then this approach would be more scalable.

To better illustrate this point in the manuscript we have changed LINE 245 to: “*Our results show a clear division by continent with the African populations, Asia/Oceania populations and South American populations forming distinct clusters. Country specific clustering was also observed even within Africa, but we were unable to identify within-country origins (Fig 2B).”*

9. **Could point eight help to narrow down the origin from the out of Africa hypothesis.**

The Otto et al database has more West African countries sampled but sampling is relatively shallow. Deeper sequencing of multiple sites within West African countries would be needed to identify founder effects of slave trade origins in South America. East Africa may be relevant for Asian origins. Again, the Otto et al. (2019) database has few east African sites.

10. **I like the evolutionary relationship to the Laverania, but why was not *P. praefalciaparum* used? It is the closest common ancestor and there is a pacbio assembly in the public domain.**

As mentioned above we have now included *P. praefalciparum* in our analyses. As well as using it in place of *P. gaboni* in the species comparison, we have replaced the comparison between *P reichenowi* and the global dataset with *P. praefalciparum*. The results differed slightly to that seen in the original analysis with the African isolates representing a similar level of ancestry to the Iranian, Thai and Papua New Guinean isolates. The South American isolates were the most divergent as was the case in the original comparison with *P. reichenowi*. We have amended this section to reflect these changes and have updated Figure 3.

11. **Different methods were used to analyse the data. But was a blast approach used? I just looked at some of the data of the authors, and a simple blast of the 3D7 domains shows the proximity of African domains.**

As described above we have now included a BLAST based approach. Whilst it performed similarly to the FFP method it was not as sensitive as the JHMM or binary t-SNE methods and we thus included it in the supplementary materials. The approach is now described in the main text at LINE 365: *“We also considered using a BLAST based distance matrix in place of the binary presence/absence matrix, prior to visualization with t-SNE but found it provided a poorer resolution of the global population structure (Fig S11)”.*

12. **I like supporting datafiles, but they need a bit of cleaning and better documentation, like table legends etc.**

We have updated the supporting text file to better document these data files including a brief description of each column in the tables provided.

13. **Personally, don't like personal communication of open access publications...**

We have removed these references.

14. **The methods should be revisited. Several terms are not defined like centroid - in which context, in the alignment? Suddenly switch from DBLa to reads. What is OTU, FFP? The link to the different program on GitHub would be useful.**

We have removed the reference to ‘OTU’ replacing it with ‘binary presence/absence matrix’. We have also defined the terms ‘centroid’, ‘FFP’ and replaced the use of ‘reads’ with ‘sequence’, ‘centroid’ or ‘DBLα type’ where appropriate. We have also added links to relevant programs in the GitHub readme.

**MINOR POINTS**

15. **There are newer references to confirm 60 var genes in Pf, like the recent papers on PacBio assemblies or the reference 34 - line 83**

Updated to included Otto et al. (2019) (LINE 89).

16. **Could you please provide a reference for the expression of var genes in early gam? Line 90 -**

References for the expression of *var* genes in early gametocytes have been provided on LINE 97 as requested.

17. **Struggle to understand line 237ff, Gabon most self-matching or divergent - is that not contradictory?**

Updated (LINE 262: *“Gabon was identified as the most divergent African population (mean = 0.61 ± 0.15) and Colombia the most divergent South American population (mean = 0.83 ± 0.04) (Fig 2C, 2D).”*)

18. **Line 309 - is it possible to distinguish between mitotic and meiotic recombination?**

No. The JHMM detects recombination per se.

19. **line 405 CIDRa1.n n?**

Updated

20. **Some figures need higher resolutions.**

We have improved the resolution of the figures as requested using the PLoS PACE online program.

21. **There are some discussion about speed. How long does it take to do the JHMM analysis? Hours, days?**

We have updated this section to indicate it takes days to run the analysis as requested (LINE 510: *“However, the method is computationally expensive and can take days to run on large datasets.”*)

## **Reviewer #2**

**Tonkin-Hill et al sought to understand P. falciparum population genetics using the DBLa domains from the var gene family. These ~400bp domains are extremely polymorphic, making them a good candidate as a ‘barcode’ to define each P. falciparum isolate. The concept has been utilised multiple times by this lab, however here they present the first worldwide analysis (albeit limited to 10 countries), using 125 ‘new’ isolates and 1133 previously published isolates. They confirmed some well-established facts: parasites from each continent are genetically distinct, parasites from South America are related to Africa where they likely came from. Interestingly, they confirm and expand a recent discovery from Otto et al that some var domains are conserved worldwide, and this is only partially explained by drug selective sweeps. This opens a new door of investigations as to what might drive this selection. The novelty of the study lies in the algorithm used to analyse similarity between DBLa types, taking into account recombinations. This improved and more biologically relevant analysis will likely be used by other labs in the future. Although I wrote ‘major concerns’ here under, I think they are relatively straightforward to address and hopefully will improve this great piece of research. Disclaimer: My expertise with JHMM, tSNE, FFP and Viterbi algorithm is limited.**

**MAJOR**

1. **Fig1a explains very clearly why the previous method (PTS) can be problematic. Could you make another cartoon that describes the JHMM principle used in this study? Also, a flowchart would help to understand the pipeline used for the analysis.**

We have amended Figure 1 to include both a graphic to help explain the JHMM approach and a simplified version of the pipeline used.

2. **About Fig 3. According to Larremore et al 2015, there are 94 DBLa domains from P reichenowi. If I understand correctly, only about ~1% of these domains match P.f. DBLa in specific countries. Considering the low number of P reichenowi DBLa sequences, is it relevant to compare them against each individual country (Lines 287 to 291)?**

At the request of reviewer 1 we have now replaced *P reichenowi* with *P praefalciparum* in this analysis. We realize that our use of the word ‘matching’ can be slightly misleading and we could have made the description of this section clearer. The JHMM approach infers the probability of matching every base from the query sequence with every base in the target database. These bases are not required to be identical. In this case, after accounting for the number of isolates and sequences sampled from each country, we infer the expected proportion of sequence that matches each country across all bases.

For example, we may estimate that a 6% likelihood that the first base in a domain matches with African isolates, 2% with Asian isolates and 92% to other *P. praefalciparum* sequences. This is then calculated for every base in all the *P. praefalciparum* domains to give the overall estimated proportion. Thus, in this case it would not be 92% of the domains but 92% of the inferred ancestry.

In the analysis for Figures 1 and 2, after normalization, rare and diverged types drive more of the signal as they are more specific to a country or species and thus more informative. In the comparison between the *P. praefalciparum* and global dataset we normalise at the type level ensuring each DBLα type in the *P. praefalciparum* genome contributes equally to the final proportions. This is similar to ignoring the overall prevalence of each DBLα type which compensates for the very large differences in the number of isolates, given we are comparing only one *P. praefalciparum* genome.

To make this clearer and to update the analysis to focus on *P. praefalciparum* this section at LINE 314 now reads: *“To investigate the emergence and adaptation of P. falciparum in human populations, we used the JHMM to compare a P. praefalciparum isolate [5] against all other P. falciparum isolates in our DBLα type dataset. The JHMM model estimates the likelihood of a match between every base in each P. praefalciparum DBLα domain and every other DBLα domain sequence including the other P. praefalciparum domains. After controlling for the number of isolates and DBLα types sampled in each country, the estimated base levels probabilities were aggregated to give the estimated matching proportions at the country level. Unlike in the previous comparisons, where we normalized these proportions at the isolate level, here we normalized at the level of each DBLα type in the P. praefalciparum genome. This is equivalent to ignoring the overall prevalence of each DBLα type, which compensates for our analysis of only a single P. praefalciparum repertoire.*

*Fig 3 indicates that 81.5% of the estimated ancestry proportions for the P. praefalciparum DBLα types is found in other types within the P. praefalciparum repertoire. This is expected as it is a different species and thus the DBLα domains have diverged significantly from those seen in P. falciparum. However, 18.5% of the ancestry of P. praefalciparum DBLα domains matched those of P. falciparum DBLα types from field isolates. This is a similar proportion to that found in an analysis of P. reichenowi versus P. falciparum using laboratory clones [43]. We identified similar relationships between the P. praefalciparum types and both the African and Asian isolates (Fig 3). However, South American isolates appear more divergent representing only 3.67% of the inferred ancestry. The smaller proportions in South America could be attributable to stronger bottlenecks during introduction*.”

3. **My understanding of the algorithm behind tSNE is limited, but I disagree with line 254, on presented figures (Fig S3 & S4), the clustering by country is not evident. On S3, half Thai isolates cluster within Uganda. Iran isolates are within the South America isolates (contrasting with Fig 2B where they were closer to Asia). On S4, Peru and PNG isolates cluster with Gabon. I agree that, biologically, we expect geography to override the asympto/uncomplicated classification, however that is not apparent on the tSNE analysis.**

We realise we may have worded this section too strongly. The t-SNE algorithm is not guaranteed to preserve large distances and thus the distance between clusters can vary between runs. The reduced number of isolates in Figs S3 and S4 also reduces the ability of the algorithm to identify distinguishing features and thus probably contributes to the clusters being closer together. Despite this, we believe the broad grouping by country is still evident in Figs S3 and S4. The Thai isolates in Fig 2B are quite close to the Ugandan ones as well as a number being intermingled with the Iranian isolates. This is also true in Fig S3 however the distances between the major groups has reduced markedly and there is certainly less distinction between the groups.

We have thus amended the relevant text at LINE 283 to read: *“Despite this, clustering by country within each disease category is still evident although the distance between the major groups is reduced and the clusters are less clearly defined (Figs S3 & S4). Some of this reduction in definition is likely to be the result of the reduced number of isolates leading to a weaker signal for the t-SNE algorithm. The t-SNE algorithm is also not guaranteed to preserve large distances and thus changes in distances between clusters cannot be easily interpreted. In addition, the JHMM inferred proportions were found to have a significant and larger effect size than disease status in the multinomial logistic regression. This coupled with the observation that the inferred proportions for Ghanaian isolates more closely resembled Ugandan isolates than the other asymptomatic isolates, suggests that the overriding signal in this dataset is due to geography.”*

4. **Also, about Fig S3, Peru is on the uncomplicated malaria tSNE plot and should not be there.**

A number of the Peruvian isolates (Iquitos, N=21) were from uncomplicated malaria cases. These isolates are referenced in Table 1 (Yalcindag et al. (2012) and Rougeron et al. (2017)) LINE 293.

5. **Line 312, comparison with previous methods. To summarize crudely, the authors are comparing their JHMM + tSNE analysis to the previous PTS + phylogenetic tree / admixture. As the plots are different, it’s not intuitive to compare the previous and current methods. In your dataset, how many reads potentially show the scenario described in Fig1a? This would help me get a feeling for the necessity of the JHMM method.**

This is best answered by considering the DBLα types that are ignored by the binary presence/absence-based analysis. In the case of the different species, none of the *P. billcollinsi* and *P. reichenowi* types are within the standard 96% identity threshold and thus using this approach would suggest that there was no relationship between the *var* genes of the different species. Fig 2B clearly indicates that the JHMM is able to resolve this issue and reproduces similar distances to those previously found using SNPs. In the global analysis 56.8% of the DBLα types are singletons using the 96% identity threshold. Thus, any analysis using the binary approach is ignoring the majority of the information provided by the DBLα sequences. Whilst we are able to see structure using the binary types with the t-SNE analysis, the mixtures provided by the JHMM are more clearly defined. This is most noticeable when comparing the inferred mixtures in the Thailand and PNG isolates in Fig 2C with the t-SNE plot (Fig 2B). In the t-SNE plot, whilst some clustering is evident the separation is far less clear than that provided by the mixture-based analysis.

To better highlight the loss of information in the binary level analysis and thus motivate the JHMM approach we have amended LINE 209 to read: “*It is important to note that because the inter-species sequence diversity is high, this comparison would not have been possible using the commonly-used pairwise sequence identity threshold of 96%, as none of the DBLα types matched between P. reichenowi, P. billcollinsi, and P. falciparum within the 96% threshold.”*

And added the following text at LINE 254 to the Global section: *“As 18,578 of the 32,682 (56.8%) DBLα types were only seen once (i.e. singletons), any approach based on binary presence/absence will ignore the majority of the sequence data available, reducing the sensitivity of the method. In contrast, the binary approach of the JHMM accounts for all the available sequences.”*

6. **The Supplementary text is a bit confusing because the PCA is missing in Fig S5 and S6. Also, when Fig S9 is cited, it should be Fig S8.**

The supporting figures have now been included and referenced correctly in the text.

7. **Finally, on the tSNE plot, have you labelled the Uganda samples with the 6 regions of origin? I presume there is no ‘intra-country’ cluster?**

We did not identify strong intra-country clustering in Ugandan samples and thus restricted the labels to the country level. We have added the following sentence at LINE 255 to make this clearer: *“Country specific clustering was also observed even within Africa, but we were unable to identify within-country origins (Fig 2B). This clustering is unlikely to be the result of recent transmission as these isolates were taken from multiple geographically distant sites within countries over a period of years (Table 1).”*

8. **Line397. If I remember correctly the DBLa universal primers do not match type3 var genes. This would explain why they are not detected here. (Otto2019 found 680 copies of these genes in 714 isolates, so it would be very surprising if they don’t exist in the countries from this study)**

The reviewer is correct, the universal DBLα primes utilized (developed by Taylor et al. (2000)) do not match to the conserved *var3*, and this is the reason we do not detect *var3* in our DBLα type data. Rask et al. (2011) has shown that the distinct DBLα1.3 of *var3* is a DBLα-DBLζ hybrid with the N-terminal portion of the DBLα1.3 being similar to other DBLα domains, while the C-terminal half of the domain is in effect a DBLζ3 domain. We have clarified this on LINE 440: “*This was expected as the universal DBLα primers used (developed by Taylor et al. (2000) [58]) do not match the distinct DBLα1.3 domain of var3 which is a DBLα-DBLζ hybrid [6].”*

9. **Line 348-349: The most frequent DBLa types were selected as those present in > 50 isolates. However the number of isolates per country is very unevenly distributed. Does it mean that most of the highly frequent DBLa types are from Africa? Could you normalize the number of isolates per country (as in Otto 2019, for example)?**

The purpose of this section was to highlight unusually conserved *var* sequences rather than generate a representative sample from all countries. As the vast majority of *var* diversity is seen in Africa and isolates in South America tend to be more conserved, weighting by the small number of samples in some countries would lead us to focus almost entirely on types seen in South America. We realise that accounting for sampling differences is important in interpreting population structure and this is done in our use of the JHMM algorithm. However, we feel that when discussing conserved types it is of more interest to focus on those that remain conserved despite high levels of diversity. For this reason, we did not adjust for sampling differences in this section.

10. **The authors claim that they can track DBLa types to specific localities, countries and continent, thus they can be used as markers of geographic patterns. In my humble opinion it still remains to be shown that these ‘DBLa signatures’ are stable over time. From one year to the next, due to high recombination within var genes, the DBLa signature could be quite different and making it impossible to track where an isolate originates (other than the continent level). It sounds like this is what Dr Ruybal-Pesantez is currently working on in Ecuador, and I’m very much looking forward to reading about it. But as of today I believe more temporal data is needed.**

Our claim to track DBLα types to specific localities, countries and continents was proposed as a potential use of our data and method. It is based on the JHMM output that points to geographic signatures identified within types by a method that takes account of recombination. This result can be seen in Figure 2c where analysis of types within a country shows greater self-matching than to types in any other country. The geographic structure we see for Africa is particularly compelling as recombination has been occurring over thousands of years with country specific geographic signatures detectable with our method. It is incorrect to think that the geographic signatures we observe would disappear from one year to the next when they have arisen as a consequence of recombination over thousands of years in Africa and Asia-Oceania and over 500 years for South America. Furthermore, these geographic signatures that lie within types are consistent with the robust geographic structure seen with whole genome SNP analysis that shows geographic structure within and between continents.

As the JHMM method for analysis takes account of recombination, I am not sure why the reviewer has focused on the issue of stability of DBLα signatures to question our claim. When recombination occurs, the geographic signatures that lie within types will be recombined with other types from the same genome or local genomes that also have local geographic signatures. Data in Figure 2c illustrate this point statistically for types from the same vs other geographies.

Reviewer 2 has referred to “DBLα signatures” saying they may not be stable over time. We have assumed that he meant the within type geographic signatures that we have identified by JHMM as this is the subject of our paper. Stability of DBLα types within a location is a separate but related scientific question that is not the focus of our paper. However, there is plenty of evidence that stability of types exists within local populations.

The reviewer says that “more temporal data is needed”. We assume that this could only mean to determine DBLα type stability as the geographic signatures we detect within types have arisen over thousands of years. DBLα type stability is of interest but not necessary to publish this paper as it is not in the scope of the current manuscript under review. This manuscript is about JHMM analysis of cross-sectional data revealing geographic population structure of DBLα types using a JHMM method.

Stability of types is a subject to be explored further to better understand *var* population genetics. To this point, there is ample evidence of the stability of many DBLα types within country. For example, finding the same types multiple times in different hosts in a site provides strong evidence of stability of the majority of DBLα types. To this end we have shown spatial stability seen as reappearance of the same types in multiple isolates (not just those defined as global conserved types) at the within country level and at different time points in the datasets examined here. Uganda provides a striking example where we have previously published that 40.6% of types are seen more than once in 6 locations over the whole country (Ruybal-Pesantez 2017, doi: 10.1038/s41598-017-11814-9). In addition, we have previously published (Rougeron et al 2017, doi: 10.1002/ece3.3425) spatial and temporal stability over large distances and 7-year time frames at the within continent level in South America.

We have changed LINE 509 in the manuscript to clarify that we are speaking of geographic signatures within DBLα types as detected by JHMM when making the abovementioned claim: *“**We have demonstrated the power of using the JHMM method to investigate var gene populations. However, the method is computationally expensive and can take days to run on large datasets. With the increasing size of data sets and for this approach to be useful in larger geographic surveillance studies, its computational performance will need to be improved. A large database of geographically assigned DBLα types analyzed by the JHMM for geographic signatures would be the goal to underpin a cost-effective surveillance method in a manner similar to influenza global surveillance with hemagglutinin sequences. “*

**MINOR**

11. **In general, for each supplementary figure, please make sure to indicate what dataset and what method was used in the figure legend. A workflow of the analysis would help.**

We have added a simple workflow to Figure 1. The exact commands used in each stage of the analysis are given in the GitHub R markdown files. We have also improved the figure legends in the supporting material.

12. **Line 100: I’m not sure if Ref20 is relevant here?**

We think that perhaps the reviewer was referring to Ref 20 on LINE 89 (i.e. Larremore et al. (2015)). If this is correct, we agree with the reviewer and have removed this reference (LINE 112).

13. **The colour code (currently alphabetical) is not intuitive, it would be better to arrange it by geography.**

We have rearranged the legends by geography as requested in all required Figures.

14. **Fig1a, line 172. This might be a question of semantic, but to me the cartoon could result from a single recombination event (non-homologous translocation with reciprocal exchange)**

We have updated the Figure legend.

15. **Line 207 and Fig 2A. I’m confused as why some isolates may have 19 or fewer DBLa because in the Methods (Line565) it says that isolates with fewer than 20 sequences are removed?**

We worded this poorly and have replaced the text, see LINE 227: *“t-SNE plot constructed from the binary presence/absence matrix of DBLα types. Colors represent countries, each isolate is represented by a single point. Isolates with less than 20 DBLα types have been excluded.”*

16. **Line 246, the paragraph investigates Multiplicity Of Infection as a confounding factor and mentions the number of DBLa types to infer the number of strains present in an isolate. Could you indicate that inferred MOI in Table 1? I’m surprised that the number of DBLa types is not associated with country of origin, we typically expect higher MOI in African countries than elsewhere.**

We realise we could have made this clearer. We do not actually infer the MOI and rather use the overall number of DBLa types as a proxy. Although there is a correlation between the number of types and country, the regression indicates that the inferred ancestry proportions are a much better predictor of an isolate’s country than the number of DBLa types. This suggests that the dominating signal in the estimate of proportions is not being driven by the number of DBLa types in an isolate. This is also evident by comparing the overlap in Fig S1, which is not seen in the t-SNE plot.

To make this clearer in the text we have added the following text to LINE 273: *“While we do not infer the multiplicity of infection for each isolate directly, the number of DBLα types was found to be a weaker predictor of an isolate’s country of origin than the inferred mixture proportions. After accounting for the inferred mixture proportions, the number of DBLα types was not found to be significantly associated with an isolate’s country of origin (all p > 0.97). This is consistent with the large overlap in the distributions of the number of DBLα types by country shown in Fig S1. If the observed signal was driven entirely by the overall number of types we would expect to see similar levels of overlap in both the t-SNE plot (Fig 2B) and the JHMM mixtures (Fig 2C), which is not the case.”*

17. **Line 382: Please rephrase ‘Every high-frequency type was seen in at least one Asian or African country, except for 19 types that were only seen in African countries.’**

LINE 425 now reads: *“Of the 100 high-frequency DBLα types, 81 were seen in both Asia and Africa, with 19 types that were found only in African isolates.”*

18. **Line410. What var gene is ‘NTSB3-DBLα0.4-CIDRα6-DBLβ5-DBLγ10-DBLδ6-CIDRβ2 ‘? Is it conserved in 3D7? If so what’s its ID?**

The var gene is conserved in many different isolates and so has many accessions, the one provided in the paper was QG0292-C-g1520. This conserved DBLα tag is not present in 3D7, the most similar sequence in 3D7 is only 39% identity.

19. **Line 495: Was the sample collection approved by a local Ethics Committee? (for the new sample collections)**

Ethical approval was obtained from the local Ethics Committees for the samples collected in Peru, Thailand, and Iran. The references for these samples have been provided in the manuscript and in Table 1 (i.e. for Peru see Branch et al. (2011), for Thailand see Pumpaibool et al. (2009) and Yalcindag et al. (2012), and for Iran see Yalcindag et al. (2012)). In addition, approval was obtained from the Ethics Committees at New York University (New York, US) and the The University of Melbourne (Melbourne, AU) to undertake the necessary sequencing/analysis on these samples as provide by our co-authors O. Branch and L. Ruiz-Mesia (Peru), T. Pumpaibool and P. Harnyuttanakorn (Thailand), and S. Zakeri (Iran). We have amended the text in LINE 294: *“P. falciparum isolates previously collected from these locations were sequenced in the present study (see Methods and the references provided for all study details).”* and LINE 643 “*All DBLα sequence data included in this global study were obtained from P. falciparum isolates previously collected as described in Table 1 [31, 36, 39-41] except for the P. falciparum isolates from Peru (Zungarococha/Mazan) [46], Thailand [31, 47], and Iran [31] that were previously collected but were sequenced for the present study. “*

20. **Line 536: Why using the term ‘read’ when talking about a translated sequence?**

We have removed this term.

21. **Line 552: According to Table 1, Venezuela is the smallest dataset, not Colombia.**

Updated

22. **Line 568: ‘… a threshold of 2 DBLa types’.**

Updated

23. **Citation format is not consistent (e.g. line 103)**

We have corrected the references to be consistent.

24. **Something went wrong with many of the references. Some include the word ‘ [Internet]’ and the URL.**

We have corrected the references.

25. **Fig 4. What is the difference between grey and black dots? Could you indicate UpsA / non-upsA groups?**

All the points should be black. This appears to be an issue with the image quality. We have recreated this figure to hopefully resolve this problem. We have also added the UPS groups to Fig 4.

26. **Fig S5. The title of that figure is the exact same as Fig 2C so it’s a bit confusing. I’m not sure if FigS5 is really necessary.**

We have changed Fig 2C and Fig S5 titles to be clearer.

27. **Fig S6: the legend is confusing, please rephrase.**

We have removed the legend and tried to improve this figure and its description.

28. **Fig S7. In the legend, the description of the two plots is inverted. Is the recombination hotspot in the middle of the DBLa domain corresponding to the relatively conserved “REDWW” domain?**

We have fixed the description. The hotspot is just upstream of homology block 5 and thus the REDWW sequence.

29. **Fig S14. X and Y axis labels, replace ‘gene’ by ‘DBLa type’.**

Updated Fig S14.

30. **Fig S15. I presume the DBLa type order is the same in panel A and B? Please indicate. Actually, there are two Figures S15.**

Updated

31. **Fig S16 named S15 and line 569 : ‘some clustering by coverage (x-axis) is evident’), I can’t see it on the plot.**

We have removed this line.

32. **Table S1. Columns C and S have no header. SampleID missing in D7. Please indicate what the bold / blue /red colour scheme corresponds to.**

We have added column headers and provided a description of the colors in the supplementary text.

33. **What is “FCR3var3” in column C? Because in the main manuscript it says that type3 var were not part of the top100 DBLa types.**

This was not *var3* but was a reference to obsolete classifications that appear to be 3’ recombinant progeny of var1. The column had been retained inadvertently when the relevant text was removed from the manuscript. The column has now been removed from the table.

34. **Did you use the 15 PacBio genome assemblies from Otto2018? This might help identify chromosomal location of some of the conserved DBLa.**

We did not use the 15 assembled genomes but instead used the much larger assembled *var* gene database. We queried the global *var* database with our frequently detected DBLα sequences to determine whether they indeed marked globally conserved genes, which some indeed appear to do. It would be interesting to investigate the genomic location of the conserved var genes but we believe that it is beyond the scope of this paper which is focused on global *var* gene population structure rather than genome organization.

35. **SuppDataFile1.xlsx needs some information about what each spreadsheet is.**

We have removed the redundant spreadsheets and added a description of each column in the supplementary text.

36. **SuppDataFile3.txt, the header line is missing.**

Updated

37. **Does the file**

**‘**[**https://raw.githubusercontent.com/gtonkinhill/global_var_manuscript/master/data/combined_all_454_noPOR.fasta**](https://raw.githubusercontent.com/gtonkinhill/global_var_manuscript/master/data/combined_all_454_noPOR.fasta)**’ contain all DBLa types used in this manuscript?**

This file contains all reads with exception of those from PNG and ape species. Both of these are also included in the data folder but can also be retrieved via from the cited publications in the text.
